# Outcome and Complications of Frontal Sinus Stenting: A Case Presentation and Literature Review

**DOI:** 10.1155/2020/8885870

**Published:** 2020-08-25

**Authors:** Mohammed A. Khan, Waleed A. Alshareef, Osama A. Marglani, Islam R. Herzallah

**Affiliations:** ^1^Ear Nose and Throat Department, Head and Neck and Skull Base Center, King Abdullah Medical City (KAMC-HC), Makkah, Saudi Arabia; ^2^Department of Ophthalmology & Otolaryngology, Umm Al-Qura University, Mecca, Saudi Arabia; ^3^Department of Otorhinolaryngology, Head & Neck Surgery, Faculty of Medicine, Zagazig University, Zagazig, Egypt

## Abstract

**Introduction:**

Frontal sinus surgery remains challenging to manage because of its complex anatomy and narrow outflow tract. A number of studies suggest the success of frontal sinus stenting to reduce postoperative complications in endoscopic frontal sinus surgery. However, failure and complications of frontal sinus stenting may occur.

**Method:**

We present a case of frontal sinus stenting with migration of the stent and erosion of the lamina papyracea together with a granulomatous reaction around the stent. PubMed and Medline search was also conducted to study the current evidence on frontal sinus stenting benefits and complications.

**Results:**

Still there are no guidelines or universally accepted indications for the use of frontal sinus stenting in the literature. A limited number of studies suggest the success of frontal sinus stenting to reduce postoperative stenosis in endoscopic frontal sinus surgery. However, failure and complications of frontal sinus stenting may occur. Infection, pain, edema, and stent obstruction may also occur. Our case report also highlights the potential of orbital complications as well as the consequences of inducing a granulomatous reaction.

**Conclusion:**

The value of frontal sinus stenting is still a subject of debate. Complications of frontal sinus stenting are not uncommon and thus necessitate regular follow-up.

## 1. Introduction

Because endoscopic surgery is now commonly used to manage nasal pathologies, many endoscopic techniques for the management of simple and complex frontal sinus diseases have been developed. Frontal sinus disease remains challenging to manage because of the complex anatomy and narrow outflow tract [1]. Failure of frontal sinus surgery has been frequently reported. [2] The most common causes of failure in the frontal recess are remnant frontal recess cells, a retained uncinated process, middle turbinate lateralization, and postoperative stenosis of the frontal sinus outflow due to formation of scar tissue, synechiae, or osteoneogenesis [2].

A limited number of studies suggest the success of frontal sinus stenting to improve outcome in endoscopic frontal sinus surgery [3–7]. However, failure and complications of frontal sinus stenting may occur. Infection, pain, edema, and stent obstruction were reported [8].

Still there are no guidelines or universal accepted criteria for the use of frontal sinus stenting; the use of it may depend on the surgeon's decision and the operative scenario. Hosemann demonstrated that frontal sinus outflow tract (FSOT) stenosis is more in patients with the diameter of the neo-ostium less than 5 mm, and this may be considered as an indication for stenting [1].

Double J stent, frontal sinus stent acting as a local drug-releasing system, Rains self-retaining silicon stent, doxycycline-releasing stent, and H-shaped silicon stent are examples of many types used [5, 9].

## 2. Case Report

A 16-year-old male was referred to our Tertiary/Quaternary Care Hospital, King Abdullah Medical City, in 2015 with a history of allergic fungal rhinosinusitis (AFRS) and functional endoscopic sinus surgery (FESS) one year ago. The operative report included left frontal sinus stenting using a biliary T-tube stent. The patient came to the outpatient department complaining of bilateral nasal obstruction and decreased smell sensation along with left ocular symptoms. Ophthalmic symptoms included left eye pain, discomfort, and redness without eye movement restriction.

**Figure 1:**
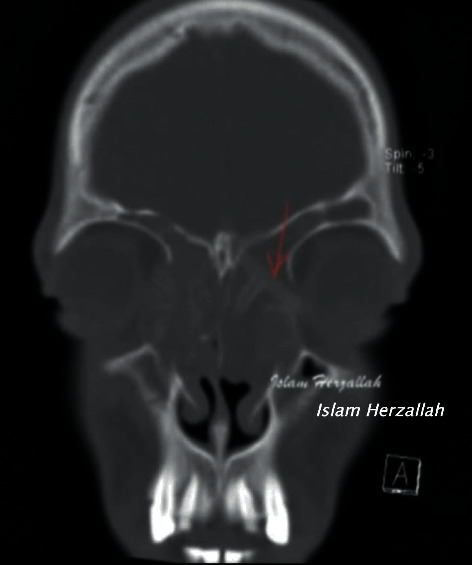
Preoperative CT scan showing migrating frontal sinus stent.

**Figure 2:**
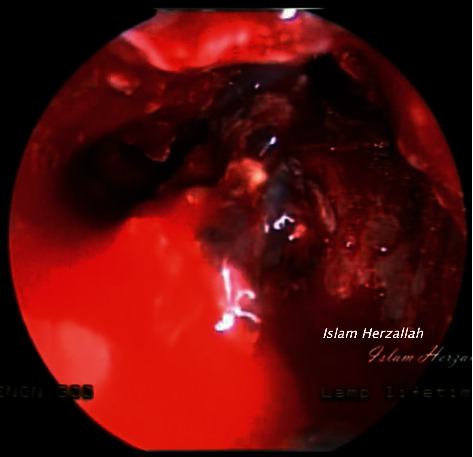
Intraoperative granulation tissue.

**Figure 3:**
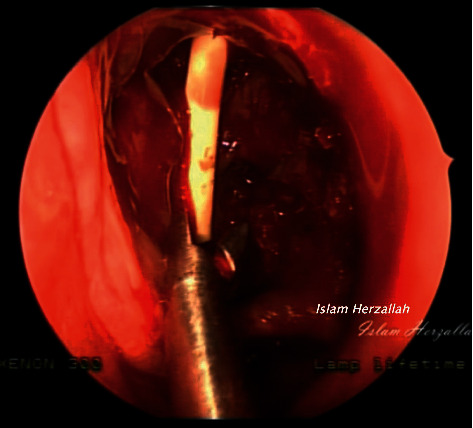
Intraoperative stent removal.

**Figure 4:**
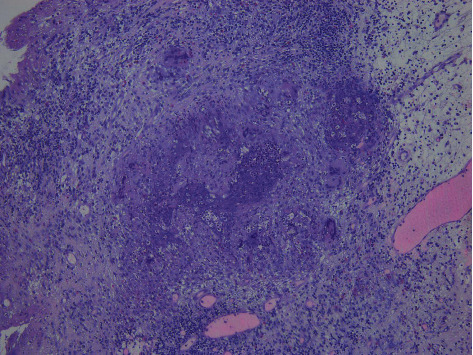
Low-power view showing granulomas and giant cells.

On examination, recurrent grade 3 nasal polyps were found bilaterally. The CT report showed a migrating frontal stent that pushes against the left lamina papyracea (Figure 1). The lamina papyracea was intact in all previous follow-up CT scans. In our institution, the patient underwent extended endoscopic sinus surgery with the Draf III procedure performed by the senior author (I.R.H.). Intraoperatively, granulation tissue was forming around the stent, which was removed, and the specimen was sent for histopathological analysis (Figures 2 and 3). The latter was initially interpreted as possible granulomatous invasive fungal sinusitis, but then proved to be foreign body granulation tissue in reaction to the stent (Figure 4).

## 3. Discussion

The effectiveness of frontal sinus stenting is still a subject of debate in the literature. A limited number of studies suggest the effectiveness of using the stent in frontal sinus surgery (Table 1) [3–5, 7]. Weber et al. showed high effectiveness in the stented group using a silicon stent versus the nonstented group: the neo-ostium of the frontal sinus was patent in 80% of the stented group evaluated by endoscopy and CT/MRT together while it was patent in only 33% of the nonstented group (Table 1) [3].

On the other hand, Banhiran et al. studied long-term effects of frontal sinus stenting by evaluating the postoperative patency of frontal ostium and the postoperative improvement in patients' symptoms among stented and nonstented groups. The results showed no statistical difference between the two groups in ostium patency and symptoms improvement (Table 1) [10].

The optimal duration of stenting the frontal sinus is unknown. Orlandi et al. demonstrated that stent can be very well tolerated postoperatively and the mean length was 31.6 months but without identifying the optimal duration [8]. One case reported a success stenting kept in place for 21 months without any complications [11]. In our case, the stent was kept in the patient for 12 months and was complicated by ocular symptoms as well as granulation tissue formation (Table 1).

In our present case report, the use of the stent was accompanied by different complications. The first complication was migration: our case report is the first describing such a complication with using the stent. The CT scan showed the migration of the stent through lamina papyracea which caused the ocular symptoms: left eye pain, discomfort, and redness.

The second complication was granulomatous tissue formation: granulomatous tissue was formed around the stent resulted in misinterpretation of the specimen histopathologically as granulomatous invasive fungal sinusitis, due to the presence of foreign body-type multinucleated giant cells, which is the characteristic finding in both conditions (Figure 4).

Other complications of the frontal sinus stent have been reported. Examples include secondary infection and hypersensitivity [12], bacterial biofilm on stent, and toxic shock syndrome [13, 14].

On examination, the patient had recurrent grade 3 nasal polyp, and obviously the presence of frontal sinus stent was not helpful in providing the appropriate irrigation to the frontal recess. Instead of keeping the patency of the ostium, the stent resulted in unwanted outcome. As well, the granulation tissue formed around the stent was histopathologically misinterpreted as a granulomatous invasive fungal sinusitis. Further studies are strongly needed to judge the effectiveness of using the stent.

## Figures and Tables

**Table 1:** Summary of studies showing the effectiveness, optimal duration, and complications of frontal sinus stenting.

Title	Author	Type of study	Year	Result
Endonasal Frontal Sinus Surgery with Permanent Implantation of a Place Holder	Weber et al.	Controlled prospective	1997	80% patent FSOT in the stented group vs. 33% in the nonstented group
The Success of 6-Months Stenting in Endonasal Frontal Sinus Surgery	Weber et al.	Retrospective	2000	Long-term stenting of the FSOT prevented restenosis in revision cases but did not prevent polyp regrowth
Frontal Sinus Stenting	Rains	Prospective (no control)	2001	94% patent FSOT
Toxic Shock Syndrome Associated with Frontal Sinus Stents	Chadwell et al.	Case report	2001	TSS can be complicated by the frontal sinus stent
Evidence of Bacterial Biofilms on Frontal Recess Stents in Patients with Chronic Rhinosinusitis	Perloff et al.	Prospective	2004	Evidence of bacterial biofilms on the stent of 6 patients
Long-Term Effect of Stenting after an Endoscopic Modified Lothrop Procedure	Banhiran et al.	Controlled prospective	2006	No difference between stented and nonstented groups
Prolonged Stenting of the Frontal Sinus	Orlandi et al.	Retrospective	2009	The mean length of stenting was 31.6 months
Failed Endoscopic Sinus Surgery	Huang et al.	Retrospective	2009	Explained the causes of failed endoscopic sinus surgery
Long-Term Stenting for Chronic Frontal Sinus Disease	Hunter et al.	Retrospective case series	2010	Three cases were successfully treated with long-term frontal sinus stenting
Twenty One Months of Frontal Sinus Stenting	Ivana et al.	Case report	2012	The stent was kept in place 21 months
Double J Stent of Frontal Sinus Outflow Tract in Revision Frontal Sinus Surgery	Mansour H.	Prospective (no control)	2013	Four of the 5 patients (6 out of 7 sinuses) had a patent frontal outflow tract

## Data Availability

No data were used to support this study.
